# A rare case of intrathoracic Gauzoma

**DOI:** 10.1186/s13019-024-03264-y

**Published:** 2025-01-08

**Authors:** Taishi Adachi, Hidenao Kayawake, Hiroshi Hamakawa, Yutaka Takahashi

**Affiliations:** Department of Thoracic Surgery, Kobe City Medical Center General Hospital, 2-1-1 Minatojima-Minamimachi, Chuo-ku, Kobe, 650-0047 Japan

**Keywords:** Gauzoma, Foreign body granuloma, Less-invasive surgery, Postoperative computed tomography

## Abstract

**Background:**

Gauzoma is a foreign body reactive granuloma which is an extremely rare complication of thoracic surgery. We describe a case of a Gauzoma in which the gauzes were removed by mini-thoracotomy as a less invasive procedure, discovered incidentally after 35 years of follow-up.

**Case presentation:**

A 51-year-old man was referred to our department for hyperhidrosis treatment, and imaging studies and biopsy confirmed the diagnosis of Gauzoma. As the Gauzoma gradually grew for a few years, surgical intervention was judged necessary, and the removal of the gauzes was performed in this case. A two-stage operation in two days was required to complete the surgery. The completion of gauze removal was confirmed in the second surgery using a postoperative computed tomography (CT) scan. The patient is currently doing well 17 months after surgery, and the size of granuloma unchanged.

**Conclusion:**

We performed the removal of the gauzes as the treatment for Gauzoma. Although removing the gauze may have prevented its growth so far, careful follow-up is still needed.

**Supplementary Information:**

The online version contains supplementary material available at 10.1186/s13019-024-03264-y.

## Introduction

Gauzomas, granulomas caused by a reaction to gauze, are occasionally found in the abdominal cavity [1]. In the thoracic cavity, the rate of occurrence varies between 0.001 and 0.0001% [2]. The reported risks of Gauzomas include emergency surgery, unplanned intraoperative changes, and a high body mass index [3, 4]. We describe a case of thoracic Gauzoma discovered incidentally after 35 years, in which gauze removal was performed as a less-invasive procedure.

## Case presentation

**Fig. 1:**
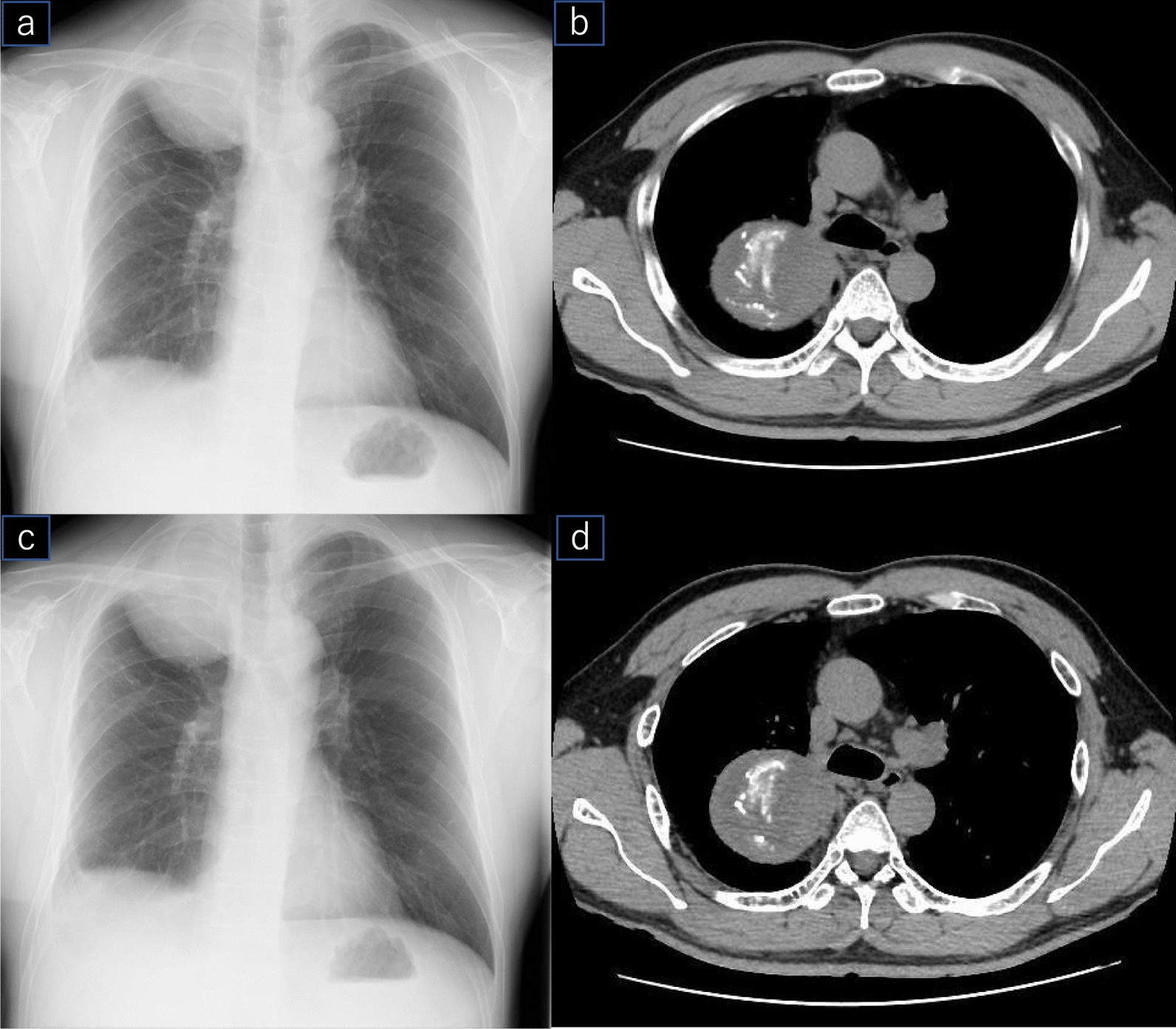
**a** On radiography, gauze fragments are not found in the mass. **b** Computed tomography reveals a gauze fragment with calcification in the granuloma. **c** Two years later, the mass has increased in size. **d** Granuloma is pressing on the trachea

**Fig. 2:**
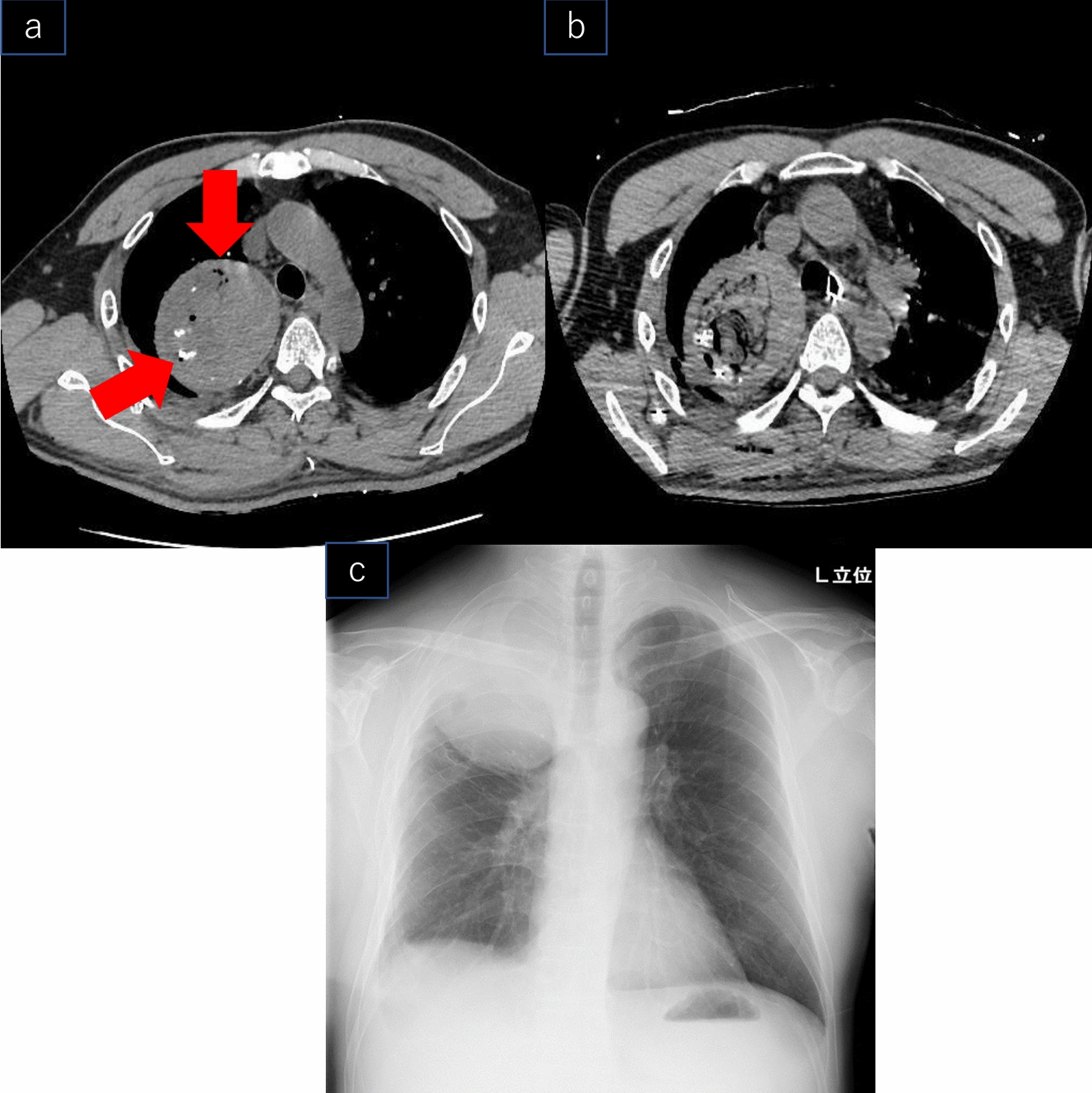
**a** Arrowhead indicates the non foreign object removal area and gauze. **b** Computed tomography reveals that the foreign object removal is completed. **c** X-ray 5 months after the operation

A 51-year-old man was referred to our institution for hyperhidrosis treatment, and was asymptomatic. Chest radiography revealed an 85 × 64 mm mass shadow in the right upper lung field (Fig. 1a). Moreover, chest computed tomography (CT) revealed gauze fragments with calcification within the mass (Fig. 1b). The gauze fibres were detected by echo-guided biopsy, indicating the presence of a Gauzoma. Since the patient was asymptomatic, only close monitoring was adopted. Two years after the initial assessment, the mass increased to 108 × 90 mm (Fig. 1c), resulting in tracheal compression (Fig. 1d). Surgical intervention was recommended, as enlargement of the gauzoma could compress the airway and potentially lead to respiratory distress. Thirty-five years prior to hospitalisation, he underwent a right open chest emergent surgery for traumatic pulmonary contusion and rib fracture at another hospital. His intraoperative records were unavailable. Since severe intrapleural adhesions were expected, the excision of gauze and tissue in the Gauzoma was performed as a less-invasive procedure in this case by mini-thoracotomy. Surgery was performed with the patient in a left lateral recumbent position, using a posterior approach (Additional file 1). We removed gauze and tissue in the Gauzoma as much as possible, confirmed the arterial pulse deep in the granuloma, and completed the surgery. Since the CT scan on postoperative day (POD)-2 showed insufficient removal of gauze (Fig. 2a), reoperation was performed on the same day. After skin closure, CT was performed to confirm the outcome (Fig. 2b).

The chest drain was removed on POD-4, and the patient was discharged on POD-8. Histopathological examination revealed no malignancies or infections. The Five months postoperatively, chest radiography showed that the granuloma had shrunk to 102 × 90 mm (Fig. 2c). Seventeen months following the surgery, the tumour size has stabilized without re-growth, and the patient is being rehabilitated well, and there were no symptoms.

## Discussion and conclusion

Previous reports have described the treatment of Gauzoma as removal with the capsule [1, 3, 5]. If left untreated, previous reports indicate a risk of infection, abscess formation, and fistula development [1, 3, 5]. Still, its necessity is uncertain, and complete removal may be difficult when the details of the initial surgery are unclear. In this case, although the details of the initial surgery remain unclear, it appears that a substantial open chest surgery was performed, indicating the likelihood of significant adhesions. Given the patient’s relatively young age and the importance of supporting an early return to daily and occupational activities, a less invasive approach was considered most appropriate. Consequently, we opted for a minimally invasive surgery to remove the gauze, as we anticipated that its removal would halt the growth of the gauzoma, despite the challenges of complete removal. Another consideration in favour of a less-invasive surgery was the patient was young and the possibility that thoracic surgery would be required in the future. Regrettably, two surgeries were required as a result. Reoperation could have been prevented by reassessment by CT immediately after surgery. In this case, we considered the possible relationship between hyperhidrosis and gauzoma. However, there are no reports suggesting an association between these conditions, and based on their respective mechanisms of onset, there is no physiological basis for a connection. Therefore, it is unlikely that hyperhidrosis contributed to the formation of the gauzoma in this case.

At 17 months postoperatively, the Gauzoma has remained stable with no increase in size. The patient will continue to be monitored regularly to follow the long-term results of this approach. In this case, although the patient was asymptomatic, surgery was performed due to the progressive enlargement of the gauzoma. Conversely, previous reports have documented cases in elderly patients where long-term follow-up revealed spontaneous shrinkage of gauzomas [6]. Surgical indications should therefore be carefully considered, taking into account the patient's age, clinical course, and the presence or absence of symptoms.

In summary, we report a very rare case of intrapleural Gauzoma. Gauzoma is a foreign body reactive granuloma, and although the removal of the gauze may have prevented its growth, careful follow-up is still needed.

## Supplementary Information

Additional file1 (Operation. A dorsal skin incision (indicated by the red line) was performed. The third rib was cut to open the patient’s chest. We ruptured the capsule and decorticated the tumour. Subsequently, we removed the two large gauzes and confirmed that the arterial pulse was deep in the granuloma. Further decortication appeared dangerous; thus, the surgery was completed.)

## Data Availability

No datasets were generated or analysed during the current study.

## Abbreviations

CT: Computed tomography
POD: Postoperative day
